# A Comparison of Different Strategies for Optimizing the Selection of Empiric Antibiotic Therapy for Pneumonia Caused by Gram-Negative Bacteria in Intensive Care Units: Unit-Specific Combination Antibiograms Versus Patient-Specific Risk Factors

**DOI:** 10.1093/ofid/ofae643

**Published:** 2024-10-30

**Authors:** Walaiporn Wangchinda, Samuel L Aitken, Megan E Klatt, Paul R Lephart, Aaron B Smith, Jason M Pogue

**Affiliations:** Department of Clinical Pharmacy, College of Pharmacy, University of Michigan, Ann Arbor, Michigan, USA; Faculty of Medicine, Siriraj Hospital, Mahidol University, Bangkok, Thailand; Department of Clinical Pharmacy, College of Pharmacy, University of Michigan, Ann Arbor, Michigan, USA; Department of Pharmacy, Michigan Medicine, Ann Arbor, Michigan, USA; Department of Pharmacy, The University of Kansas Health System, Kansas City, Kansas, USA; School of Pharmacy, The University of Kansas, Lawrence, Kansas, USA; Department of Pathology, University of Michigan, Ann Arbor, Michigan, USA; Department of Pathology, University of Michigan, Ann Arbor, Michigan, USA; Department of Clinical Pharmacy, College of Pharmacy, University of Michigan, Ann Arbor, Michigan, USA

**Keywords:** antibiogram, empiric antibiotic, intensive care unit, pneumonia, risk factor

## Abstract

**Background:**

Guidelines suggest dual antipseudomonal therapy for empiric treatment of pneumonia caused by gram-negative bacteria in intensive care unit (ICU) patients. Additionally, consideration of local susceptibility data and patient-specific risk factors for resistance is recommended for selecting optimal empiric regimens. However, data assessing how to best do this are lacking, and it is unclear whether a local susceptibility data–based or a patient-specific risk factor–based approach will better drive appropriate empiric treatment. This study aims to compare these 2 strategies.

**Methods:**

This retrospective study was divided into 2 periods. In period I, gram-negative respiratory cultures from ICU patients were used to develop unit-specific combination antibiograms, and individual patient charts were reviewed to assess the impact of risk factors on antimicrobial susceptibility to develop a risk factor–based treatment algorithm. Optimal empiric regimens based on these 2 strategies were then defined. In period II, these regimens were hypothetically applied to patients to compare rates of appropriate empiric therapy and overuse by the 2 methods.

**Results:**

Risk factor–based regimens had a higher appropriateness rate compared to regimens derived from antibiograms (89.9% vs 83.7%). Additionally, applying antibiogram-based regimens resulted in a higher prevalence of antibiotic overuse than a patient-specific risk factor–based approach (69.8% vs 40.3%), with excess overuse driven by a higher frequency of unnecessary use of combination therapy.

**Conclusions:**

Both strategies provided high rates of appropriateness in empiric antibiotic selection. However, the patient-specific risk factor–based approach demonstrated a higher rate of appropriate therapy and offered advantages in reducing rates of unnecessary combination therapy.

## Teaser

Both unit-specific combination antibiograms and patient-specific factors help improve the appropriateness of empiric antibiotic selection for intensive care unit pneumonia. However, the patient-specific risk factor approach is comparatively more effective, leading to higher rates of appropriate therapy and less antibiotic overuse.

## Graphical Abstract

**Graphical abstract:**
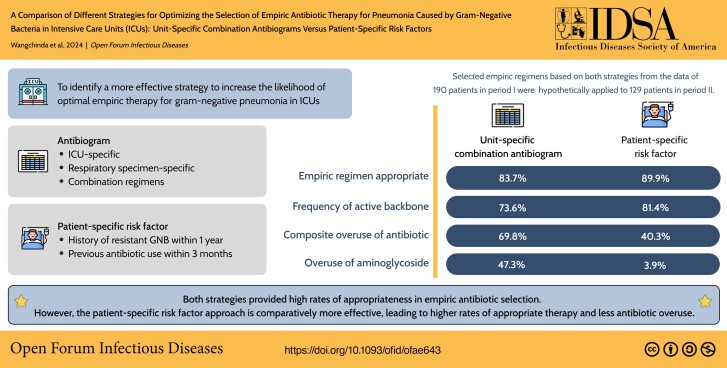
This graphical abstract is also available at Tidbit: https://tidbitapp.io/tidbits/a-comparison-of-different-strategies-for-optimizing-the-selection-of-empiric-antibiotic-therapy-for-pneumonia-caused-by-gram-negative-bacteria-in-intensive-care-units-icus-unit-specific-combination-antibiograms-versus-patient-specific-risk

Pneumonia, particularly hospital-acquired pneumonia and ventilator-associated pneumonia (HAP/VAP), is a common infection in intensive care units (ICUs) and is associated with high morbidity and mortality [1–3]. Inappropriate empiric antibiotic treatment, meaning regimens lacking in vitro activity against causative pathogens, is the most important modifiable risk factor contributing to poor outcomes [4]. Given this impact on patient outcomes, and the high rates of antimicrobial resistance to antipseudomonal β-lactams demonstrated in gram-negative bacteria causing pneumonia in ICUs [5], it is common practice to administer 2 antipseudomonal agents empirically to such patients to increase the likelihood of providing an in vitro active therapy. Indeed, this strategy is guideline-recommended in ICU patients with pneumonia who have risk factors for multidrug-resistant (MDR) pathogens, in those who reside in units with high rates of antibiotic resistance, and in patients who are critically ill [6–8]. While treatment guidelines recommend incorporating local epidemiology and patient risk factors for antimicrobial resistance into empiric treatment decisions to increase the likelihood of active empiric therapy, little guidance for how to do this is provided. This lack of clear guidance is of concern given the risks of both over- and undertreatment with standard dual-antipseudomonal regimens. Therefore, more optimal strategies to promote appropriate and optimal empiric antibiotic therapy are needed.

One common strategy to increase the probability of providing appropriate empiric treatment is developing local antibiograms based on common pathogens and using their results to inform treatment. The utility of antibiograms can be further enhanced by stratified analyses of pathogens based on hospital units and/or specific disease states to provide a more precise assessment of antibiotic susceptibility. Comparative analyses between ICU-specific and hospital-wide antibiograms demonstrated that *Pseudomonas aeruginosa* isolates exhibit higher rates of antibiotic resistance in ICUs when compared to general hospital units [9–11], and analyses of antibiograms from different sources indicate lower antimicrobial susceptibility rates for respiratory isolates compared to blood isolates [11]. Therefore, limiting antibiograms that inform empiric ICU pneumonia guidance to gram-negative respiratory isolates from that unit has the potential to enhance the appropriateness of empiric therapy [12]. Moreover, construction of combination antibiograms can further hone empiric therapy recommendations by providing crucial information on the percentage of isolates susceptible to different potential second antibiotics in the setting of resistance to a β-lactam backbone [11, 12].

However, there are concerns with broadly applying unit-specific combination antibiogram-based regimens to all patients. This approach may increase the risk of overtreatment since not all patients in the ICU with pneumonia require such aggressive empiric coverage. For example, a previous study demonstrated that early-onset HAP in ICU patients may be caused by *Haemophilus influenzae* or *Moraxella catarrhalis*, which are susceptible to more narrow-spectrum antibiotics [13]. The use of broad-spectrum combination antibiotics in such patients may not be necessary and may put the patients at risk for adverse events or the further development of antimicrobial resistance. Additionally, combination antibiograms will neglect to provide adequate coverage for infrequently encountered drug-resistant pathogens, even in patients with a significant risk for such a pathogen.

These limitations of using unit-specific combination antibiograms suggest the potential advantages of developing more individualized empiric regimens based on patient-specific risk factors. Significant risk factors for MDR gram-negative pathogens in respiratory cultures described in previous studies include prior colonization or infection by a MDR organism [14, 15], prior antibiotic use [16, 17], prolonged hospitalization [18–22], and patient's location prior to admission [23]. Evaluating these risk factors in individual patients is likely to improve the appropriateness of empiric antibiotic therapy. It may also help reduce unnecessary broad-spectrum combination antibiotic use in low-risk patients while identifying those who may benefit from empiric use of novel antibiotics if at risk of being infected with a pathogen resistant to traditional regimens. However, there has been limited assessment of the relative importance of each of these potential risk factors and how to effectively integrate them into clinical decision-making.

This study was therefore designed to overcome these data gaps with 2 main objectives to optimize the selection of empiric antibiotic therapy for gram-negative pneumonia in ICUs. First, this study aimed to identify which of the previously described risk factors are key predictors of antibiotic resistance in gram-negative respiratory isolates. The second objective was to use these data to develop empiric antibiotic treatment strategies based on both a combination antibiogram-based and a patient-specific risk factor–based approach and then hypothetically apply these regimens to patients to evaluate which approach is a better driver of appropriate and optimal empiric antibiotic therapy.

## METHODS

This retrospective observational cohort study was conducted at Michigan Medicine, a tertiary care health system in Ann Arbor, Michigan. The study was approved by the Institutional Review Board at the University of Michigan (HUM00226380). Written informed consent was not required because of the retrospective nature of this study. The study was divided into 2 time periods, calendar year 2021 and calendar year 2022. Microbiological data and the presence/absence of key risk factors for antimicrobial resistance were analyzed as described below in time period I (2021) to develop empiric antibiotic treatment regimens for treating gram-negative pneumonia in 2 different ICUs (medical ICU [MICU] and surgical ICU [SICU]). These regimens were then hypothetically applied to patients in period II (2022) to determine the comparative appropriateness of regimens driven by local antibiograms and patient-specific risk factors.

### Period I

#### Development of Combination Antibiograms

The microbiological database was queried from the University of Michigan microbiology laboratory to identify all respiratory cultures (ie, sputum, tracheal aspirate, and bronchoalveolar lavage) that were positive for gram-negative bacteria from the MICU and SICU from 1 January through 31 December 2021. Only the first culture positive for a given gram-negative pathogen for each individual patient over the time period was included, and all unique gram-negative organisms isolated were incorporated into antibiogram construction. Minimum inhibitory concentrations were tested with the Sensititre automated system and antimicrobial susceptibilities were interpreted using the 2022 Clinical and Laboratory Standards Institute M100 [24]. The commonly used antipseudomonal agents, including cefepime, piperacillin-tazobactam, meropenem, levofloxacin, ciprofloxacin, amikacin, gentamicin, and tobramycin were assessed. Intermediate isolates were categorized as resistant for this analysis. If susceptibility testing was not performed for a pathogen/drug combination, susceptibility was imputed based on the organism's usual susceptibility profile, the consideration of intrinsic resistance, cross-resistance between antibiotic classes, and the prevalence of susceptibility in national surveillance data or epidemiologic studies as described in Supplementary Methods 1. Two combination antibiograms were then constructed, 1 for each unit (ie, MICU and SICU), by assessing the activity of β-lactam monotherapy, and β-lactams with their potential combination partner agents (ie, aminoglycosides and fluoroquinolones). Isolates were considered susceptible to a combination antibiotic regimen if they displayed in vitro susceptibility to either of the 2 antibiotics.

#### Identification of Key Risk Factors for Resistance in Gram-Negative Organisms in Respiratory Cultures

The electronic medical records of all patients with included index cultures in 2021 were then reviewed to assess the presence or absence of risk factors occurring prior to the index culture. Risk factors collected for analysis included a previous positive culture with a gram-negative organism resistant to at least 1 traditional antipseudomonal β-lactam that is guideline-recommended for empiric therapy for ICU pneumonia [6–8] (ie, cefepime, piperacillin-tazobactam, meropenem, or imipenem) within 1 year, previous antibiotic exposures within 3 months, admission from an outside healthcare facility, and a prolonged ICU stay (≥5 days prior to index culture). Sensitivity analyses were also performed to compare results for other “at risk” time frames for previous antibiotic use and microbiological history. To identify key risk factors, the differences in cumulative susceptibility percentages of gram-negative respiratory isolates between patients with or without each individual risk factor were assessed. If a patient had >1 gram-negative organism in their index culture, the most resistant result for each antibiotic was selected in the analysis.

#### Development of Empiric Regimens

For empiric regimens based on combination antibiograms, the regimen selections were based on the unit from which isolates were collected. For empiric regimens based on patient-specific risk factors, patients were stratified into subgroups according to the presence or absence of key risk factors and empiric regimens were selected for patients within each subgroup. Subsequently, an algorithm for empiric treatment regimens was created.

Empiric antibiotic regimens included either monotherapy with a single antipseudomonal β-lactam or combination therapy with an antipseudomonal β-lactam paired with either an aminoglycoside or fluoroquinolone. The selected empiric regimens were chosen to achieve expected antibiotic coverage for at least 85% of patients. This threshold was both considered clinically reasonable and within the acceptable range reported in a survey study on empiric antibiotic treatment thresholds for serious bacterial infections [25] and was selected to balance appropriateness with minimizing antibiotic overuse. Additionally, general antimicrobial stewardship principles were followed in their creation (see Supplementary Methods 2).

### Period II

#### Hypothetical Application of Empiric Antibiotic Regimens to Patients in the Year 2022

Following the same process as period I, all first nonduplicated gram-negative respiratory isolates from patients in the MICU and SICU from 1 January through 31 December 2022 were identified. Subsequently, selected empiric regimens based on both approaches from period I were hypothetically applied to patients isolates in period II. For the patient-specific risk factor approach, the electronic medical records were individually reviewed to identify the presence or absence of key risk factors, and the empiric regimen was selected for patients in each subgroup based on the risk factor–based treatment algorithm.

#### Comparisons of Empiric Regimens Guided by Each Approach

Appropriateness and overuse of the selected empiric antibiotic regimens guided by the patient-specific risk factor and unit-specific combination antibiogram approach were then compared. The empiric regimen was considered appropriate if the isolated gram-negative bacteria were susceptible to at least 1 empiric antibiotic. For patients who had polymicrobial respiratory cultures, each gram-negative bacteria had to be susceptible to at least 1 antibiotic for an empiric regimen to be considered appropriate. The frequency in which the β-lactam antibiotic backbones in empiric regimens displayed in vitro activity was also assessed. Overuse was defined as unnecessary use of carbapenems when a narrower spectrum β-lactam (ie, cefepime or piperacillin-tazobactam) could have been utilized or a novel β-lactam was administered when a traditional agent (ie, a carbapenem, cefepime, or piperacillin-tazobactam) would have provided appropriate coverage. Addition of a fluoroquinolone or an aminoglycoside when the β-lactam backbone was active was also considered overuse. Both composite (any of these antibiotics) and individual agent overuse were analyzed. A selected empiric treatment could meet criteria for both appropriate and overuse.

As there was debate surrounding the selection of the second agent utilizing the antibiogram-based approach (eg, weighing slightly increased coverage vs the collateral damage of routine fluoroquinolone use), a sensitivity analysis was also conducted on regimens that used alternative second combination agents with higher susceptibility to assess how these changes to the second agent would have impacted the results.

## RESULTS

### Period I

The analysis of period I included 221 unique gram-negative respiratory isolates from 190 patients. The frequency of gram-negative bacteria among these 221 isolates is provided in Supplementary Results 1. The most common gram-negative bacteria isolated was *P aeruginosa* (25.3%), followed by *Escherichia coli* (14.9%), *Stenotrophomonas maltophilia* (12.7%), *Klebsiella pneumoniae* (12.2%), and *Enterobacter cloacae* (8.6%).

#### Development of Empiric Regimens Guided by Unit-Specific Combination Antibiograms

Of 221 index isolates included, 152 isolates were from the MICU and 69 isolates were from the SICU. Combination antibiograms specific to each unit are presented in Table 1. Overall, MICU isolates exhibited slightly higher susceptibilities to both single and combination agents compared to the SICU isolates. However, none of the single β-lactam agents achieved a susceptibility rate ≥85% in either of the ICUs. Based on susceptibilities and the stewardship principles applied, cefepime with tobramycin was selected for MICU, whereas cefepime with levofloxacin was selected for SICU. Both regimens covered 88% of the isolates in each ICU.

**Table 1.** Combination Antibiograms for Gram-Negative Isolates Recovered From the Medical and Surgical Intensive Care Units

Ward and Agent	Isolates Susceptible, % by Agent					
	Monotherapy	Amikacin	Gentamicin	Tobramycin	Levofloxacin	Ciprofloxacin
MICU (152 isolates)						
Cefepime	76	90	88	88^a^	90	82
Meropenem	84	89	88	88	96	86
Piperacillin-tazobactam	66	87	86	86	90	78
Levofloxacin	82	…	…	…	…	…
Ciprofloxacin	72	…	…	…	…	…
Amikacin	86	…	…	…	…	…
Gentamicin	83	…	…	…	…	…
Tobramycin	84	…	…	…	…	…
SICU (69 isolates)						
Cefepime	73	86	83	84	88^a^	78
Meropenem	73	83	80	81	90	77
Piperacillin-tazobactam	57	83	78	81	87	75
Levofloxacin	83	…	…	…	…	…
Ciprofloxacin	71	…	…	…	…	…
Amikacin	83	…	…	…	…	…
Gentamicin	77	…	…	…	…	…
Tobramycin	81	…	…	…	…	…

Abbreviations: MICU, medical intensive care unit; SICU, surgical intensive care unit.

^a^Selected empiric regimen.

#### Frequency of Factors for Resistant Gram-Negative Pathogens and Their Influence on Susceptibility

Of the 190 patients included in time period I, 16.8% had a positive culture for resistant gram-negative bacteria within the previous year, 60.0% had a history of antibiotic use in the previous 3 months, 36.8% were transferred from an outside healthcare facility, and 42.6% had ICU stay of ≥5 days before the index culture. Rates of previous antibiotic exposure and microbiological history were similar in sensitivity analyses of different time windows for assessing exposures (Supplementary Results 2).

**Figure 1.**
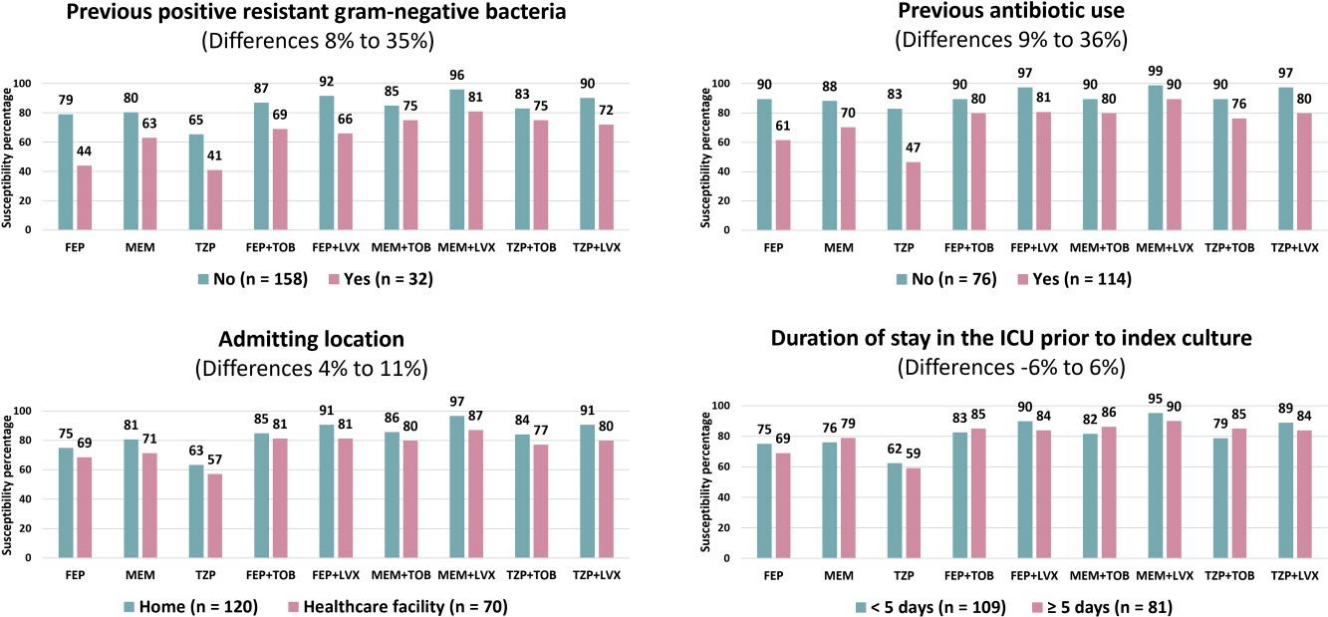
Cumulative susceptibility percentages of gram-negative respiratory isolates for patients with and without each risk factor. Abbreviations: FEP, cefepime; ICU, intensive care unit; LVX, levofloxacin; MEM, meropenem; TOB, tobramycin; TZP, piperacillin-tazobactam.

The comparisons of cumulative susceptibility percentages of various monotherapy and combination therapy regimens for index isolates from patients with and without each risk factor are demonstrated in Figure 1. Patients with a history of a positive culture for a resistant gram-negative bacteria or a history of antibiotic use had index isolates with markedly lower susceptibility rates than those without those factors, with the differences in susceptibilities ranging from 8% to 35% and 9% to 36%, respectively, for potential monotherapy and combination therapy regimens. In contrast, patients who were transferred from other healthcare facilities had a modest decrease in susceptibility rates of their isolates compared to those admitted from home, with the differences ranging from 4% to 11%. Similarly, there was no notable trend of differences in susceptibilities observed when patients were categorized based on the prolonged duration of ICU stay prior to index cultures, as the differences ranged from −6% to 6%. Indeed, when removing those with a positive microbiologic history or recent antibiotic exposure from these 2 groups, there was effectively no difference in antimicrobial susceptibility in isolates recovered from patients with or without these last 2 factors, and overall susceptibility rates were approximately 20% higher (Supplementary Results 3).

#### Development of Empiric Regimens Guided by Patient-Specific Risk Factors

**Figure 2.**
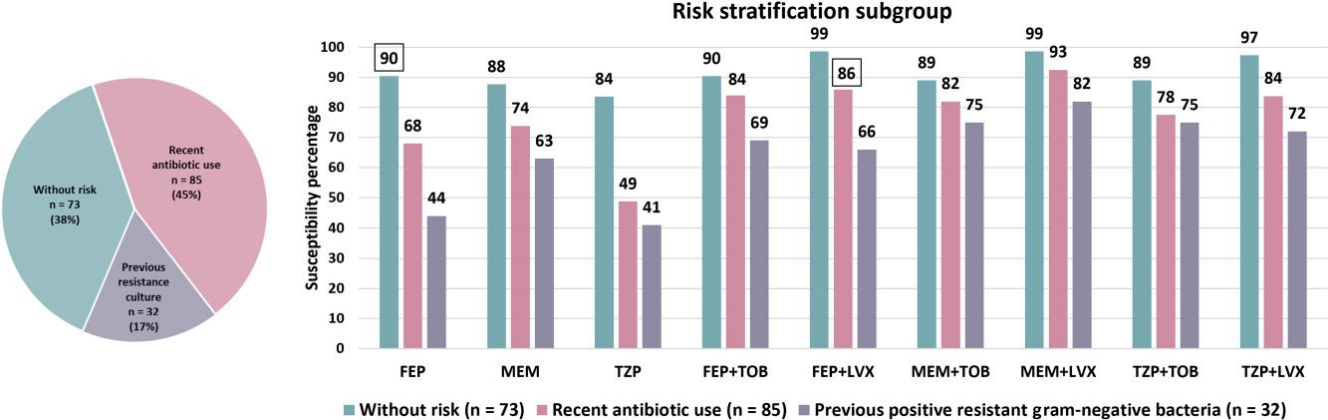
Cumulative susceptibility percentages of gram-negative respiratory isolates from patients stratified by the 2 key risk factors. □ indicates selected empiric regimen. Abbreviations: FEP, cefepime; LVX, levofloxacin; MEM, meropenem; TOB, tobramycin; TZP, piperacillin-tazobactam.

**Figure 3.**
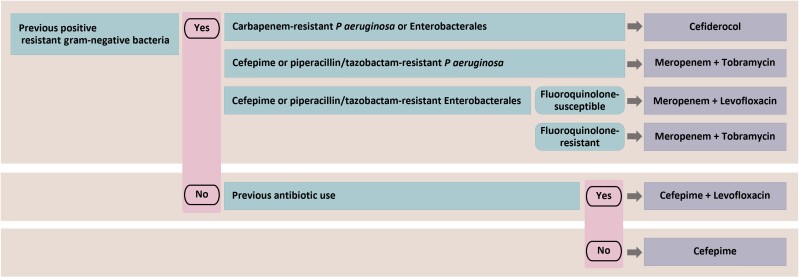
Algorithm for empiric antibiotic therapy based on patient-specific risk factors. If a patient has a history of a different gram-negative organism resistant to the algorithmic recommendation, empiric therapy should be modified to include coverage of that pathogen (eg, history of carbapenem-resistant *Acinetobacter baumannii* should include empiric coverage targeting this pathogen).

To develop an empiric regimen algorithm guided by patient-specific risk factors, patients were stratified into 3 mutually exclusive subgroups based on the presence or absence of these 2 key risk factors. The first subgroup included 32 patients with a previous resistant gram-negative bacteria isolation in the previous year, as this factor had the most substantial impact on susceptibility of the index culture. Of those who were not included in the first subgroup, the second factor considered was a history of antibiotic use in the previous 3 months, which included 85 patients. The third subgroup consisted of the 73 patients who did not have either of these 2 risk factors. The susceptibility percentages against their gram-negative respiratory isolates were compared between subgroups, as demonstrated in Figure 2 and Table 2. The algorithm of empiric regimens developed based on these risk factors is provided in Figure 3, and the rationale for their selection is described in Supplementary Results 4.

**Table 2.** Cumulative Susceptibility Percentages of Gram-Negative Isolates From Patients, Stratified by Which Previous Resistant Gram-Negative Bacteria Was Isolated

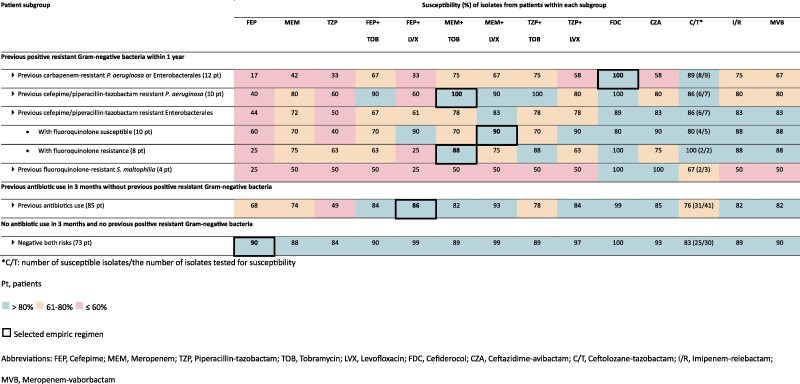

### Period II

In the analysis of period II, a total of 129 patients with 159 gram-negative respiratory isolates were identified. The frequency of gram-negative bacteria in period II was similar to that in period I (Supplementary Results 1). The frequency of empiric regimens selected for these patients after hypothetically applying the regimen based on both approaches is presented in Table 3. In the patient-specific risk factor approach, there was a wider variety of empiric regimens based on 3 β-lactam backbones: cefepime-based regimens (38.0% as monotherapy, 44.2% combined with levofloxacin, and 1.5% combined with trimethoprim-sulfamethoxazole [TMP-SMX]), meropenem-based regimens (5.4% combined with tobramycin, 3.1% combined with levofloxacin), and cefiderocol monotherapy (7.8%). Of note, the algorithm was adapted by empirically adding TMP-SMX for patients who had a history of levofloxacin-resistant *S maltophilia*. In the unit-specific combination antibiogram approach, cefepime was the backbone β-lactam in all patients and it was combined with tobramycin in 72.1% (MICU) and levofloxacin in 27.9% (SICU).

**Table 3.** Frequency of Empiric Regimens Based on 2 Strategies for 129 Patients in Period II

Empiric Regimen	Frequency of Empiric Regimens, No. (%)	
	Patient-Specific Risk Factors	Unit-Specific Combination Antibiograms
Cefepime + tobramycin	…	93 (72.1)
Cefepime + levofloxacin	57 (44.2)	36 (27.9)
Cefepime	49 (38.0)	…
Cefepime + TMP-SMX	2 (1.5)	…
Meropenem + tobramycin	7 (5.4)	…
Meropenem + levofloxacin	4 (3.1)	…
Cefiderocol	10 (7.8)	…

Abbreviation: TMP-SMX, trimethoprim-sulfamethoxazole.

#### Appropriateness and Overuse of Regimens Guided by 2 Strategies

The comparison of empiric regimen appropriateness and overuse based on the 2 strategies is presented in Table 4. Empiric regimens based on patient-specific risk factors demonstrated a higher rate of appropriateness compared to those using the unit-specific combination antibiograms (89.9% vs 83.7%). Furthermore, the risk factor–guided regimens demonstrated a higher prevalence of active β-lactam backbones (81.4% vs 73.6%). Comparing antibiotic overuse between the 2 strategies, the combination antibiogram approach resulted in a higher overuse rate (69.8% vs 40.3%), with unnecessary combination with tobramycin being the most common reason (47.3%). These results demonstrate that a patient-specific risk factor–based approach was associated with a 38% reduction in the rate of inappropriate empiric treatment, an 11% increased likelihood of an active β-lactam backbone being administered, and a 42% reduction in antibiotic overuse. In contrast, fluoroquinolone overuse was more prevalent in the patient-specific risk factor approach (31.0% vs 22.5%). The sensitivity analysis using cefepime in combination with levofloxacin as the empiric regimen in the MICU demonstrated similar results (Supplementary Results 5).

**Table 4.** Comparisons of the Appropriateness and the Overuse of Empiric Regimens Between 2 Strategies

Evaluation	Patient-Specific Risk Factors, No. (%)	Unit-Specific Combination Antibiograms, No. (%)
Empiric regimen appropriate	116 (89.9)	108 (83.7)
Frequency of active backbone	105 (81.4)	95 (73.6)
Overuse of antibiotic		
Overuse of carbapenem	6 (4.7)	…
Overuse of fluoroquinolone	40 (31.0)	29 (22.5)
Overuse of aminoglycoside	5 (3.9)	61 (47.3)
Overuse of cefiderocol	7 (5.4)	…
Overuse of TMP-SMX	0 (0.0)	…
Composite overuse of antibiotic	52 (40.3)	90 (69.8)

Abbreviation: TMP-SMX, trimethoprim-sulfamethoxazole.

Further evaluation for each empiric regimen is described in Supplementary Results 5. Most regimens provided appropriate empiric coverage in ≥85% of target patients. The most common reason for the inappropriateness was the lack of antibiotic coverage for *S maltophilia*. Cefepime monotherapy for patients without either of the 2 risk factors was associated with a high rate of appropriate coverage (92%). Cefiderocol, the only novel agent recommended in the patient-specific risk factor group, had a 100% rate of appropriateness and a 70% overuse rate.

## DISCUSSION

One major finding in this study was that a positive culture for resistant gram-negative bacteria in the previous year and a history of antibiotic use within 3 months were identified as the key drivers that significantly influence susceptibility rates in gram-negative respiratory cultures. While prolonged ICU duration prior to infection onset and admission from outside healthcare facility are commonly considered risk factors, they appear to have minimal impact on their own and may not need consideration in empiric antibiotic regimen selection for pneumonia in ICUs. However, these results should be taken in the context of the robust infection prevention practices at Michigan Medicine. Institutions with limitations in infection control resources may experience varying degrees of horizontal spread of resistant pathogens, and thus institutional exposures may be an important consideration in such environments. Furthermore, the included population had minimal recent international travel, which is an important risk factor for acquiring resistant gram-negative bacteria [26, 27]. Therefore, while our analysis identified only previous microbiology and antibiotic history as considerations for empiric therapy, these may not be applicable to all other institutions or patient populations.

Our findings demonstrated that 89.9% of empiric regimens based on patient-specific risk factors and 83.7% based on unit-specific combination antibiograms were considered appropriate. This indicates a relatively high level of appropriateness with both strategies, particularly when compared to prescribing monotherapy alone. Data from the present analysis demonstrated that if noncarbapenem β-lactam monotherapy was employed as routine empiric therapy, it would provide activity between 57% and 76% of the time, leaving many patients to receive inappropriate empiric therapy. Moreover, the rates of appropriate therapy with both assessed strategies were higher than those previously reported in general practice for critically ill patients or patients with sepsis and septic shock [28–30], suggesting that both approaches can be effective strategies to improve the appropriateness of empiric antibiotic selection.

In the comparative analysis, the patient-specific risk factor–based approach was superior to the unit-specific combination antibiogram-based approach. This was primarily because the risk factor–based approach provided more fine-tuned recommendations to individual patients, leading to improved appropriateness and a reduction in antibiotic overuse. Importantly, the risk factor–based algorithm recommended cefepime monotherapy in 38% of cases for patients without either key risk factor. This approach significantly reduced the need for combination agents but provided active therapy in >90% of patients. These findings suggest that a risk factor–based approach can effectively reduce antibiotic exposures. Additionally, despite placing broader-spectrum agents in the treatment algorithm, <10% of patients would have received meropenem, and similarly, <10% a novel antipseudomonal agent. These data suggest that a risk factor–based approach can effectively enhance appropriateness without widespread and indiscriminate use of protected antibiotics.

It is important to note that even with personalized recommendations based on patient-specific risk factors, a subset of patients (10%) still would not have received appropriate empiric treatment. This highlights the difficulties in selecting adequate empiric regimens for all patients, and the risk that atypical pathogens or unsuspected resistance mechanisms can have on patient outcomes. This underscores the importance of real-time antimicrobial stewardship interventions, such as active surveillance of respiratory cultures and utilization of rapid diagnostic tests to decrease time to appropriate therapy in the inevitable subset of patients in whom empiric regimens are inactive.

This study has several limitations. The analysis was based on retrospective data of all initial positive respiratory cultures for gram-negative bacteria obtained from ICU patients. While these cultures were typically collected for the diagnosis of pneumonia, not all of these cultures necessarily indicated true infections. Limiting the inclusion to only the first positive culture may lead to an underestimation of resistance rates because it did not account for antibiotic resistance that could develop during the hospital stay. Furthermore, the relatively small number of patients in some of the risk stratification subgroups of patient-specific risk factor approach could affect the reliability of the susceptibility percentages. Additionally, implementing a treatment pathway based on the presence/absence of recent antibiotic exposure and microbiological history requires readily identifiable documentation of these factors and, ideally, information technology solutions that easily facilitate application of these data, which may not be possible at all institutions. Finally, prospective validation of a risk factor–based approach is warranted.

In conclusion, a history of a positive culture for resistant gram-negative bacteria and previous antibiotic use were determined to be the key risk factors associated with antibiotic resistance, warranting evaluation when making empiric antibiotic treatment decisions for gram-negative pneumonia in ICU. Both patient-specific risk factors and unit-specific combination antibiogram approaches were useful and can be applied in empiric antibiotic selection. However, the patient-specific risk factor–based approach offers a higher rate of appropriateness therapy and advantages in reducing the overuse of combination agents.

## Supplementary Data

Supplementary materials are available at *Open Forum Infectious Diseases* online. Consisting of data provided by the authors to benefit the reader, the posted materials are not copyedited and are the sole responsibility of the authors, so questions or comments should be addressed to the corresponding author.

## Supplementary Material

ofae643_Supplementary_Data

## Notes

***Potential conflicts of interest.*** J. M. P. has served as a consultant for Merck, Shionogi, Melinta, Entasis, GlaxoSmithKline, and VenatoRx, and has received grants or research support from Merck, Shionogi, and Melinta. S. L. A. has served on advisory boards for Shionogi, GlaxoSmithKline, Melinta, and Basilea. All other authors report no potential conflicts.
