# Supplementary material for: A Comparison of Different Strategies for Optimizing the Selection of Empiric Antibiotic Therapy for Pneumonia Caused by Gram-Negative Bacteria in Intensive Care Units: Unit-Specific Combination Antibiograms Versus Patient-Specific Risk Factors
Source: Open Forum Infect Dis. 2024 Oct 30;11(11):ofae643. doi: 10.1093/ofid/ofae643 (PMC11584511; doi:10.1093/ofid/ofae643)
Supplement: ofae643_Supplementary_Data [file ofae643_supplementary_data.docx]

**Supplementary Methods 1. The assumption for organisms without susceptibility results**

| **Organism and antibiotic** | **Assumption made** |
| --- | --- |
| *Acinetobacter* spp. |  |
| - Ceftazidime-avibactam, imipenem-relebactam, meropenem-vaborbactam - Ceftolozane-tazobactam | - Susceptibility based on ceftazidime, imipenem, meropenem breakpoint, respectively - Susceptibility based on *P. aeruginosa* breakpoint |
| *Achromobacter* spp. |  |
| - All agents | - Susceptibility based on *P. aeruginosa* breakpoint |
| *Aeromonas* spp. |  |
| - Tobramycin | - Susceptibility based on gentamicin breakpoint |
| - Ceftazidime-avibactam, imipenem-relebactam, meropenem-vaborbactam - Cefiderocol | - Susceptibility based on ceftazidime, imipenem, meropenem breakpoint, respectively - Susceptibility based on Enterobacterales breakpoint |
| *Burkholderia cepacia* |  |
| - Amikacin, gentamicin, tobramycin | - Resistant |
| - Cefepime, piperacillin-tazobactam | - Susceptibility based on *P. aeruginosa* breakpoint |
| - Ciprofloxacin | - Susceptibility based on *P. aeruginosa* breakpoint |
| - Cefiderocol, ceftazidime-avibactam, ceftolozane-tazobactam, imipenem-relebactam, meropenem-vaborbactam | - Susceptibility based on *P. aeruginosa* breakpoint |
| *Chryseobacterium* spp. |  |
| - All agents | - Susceptibility based on *P. aeruginosa* breakpoint |
| *Cupriavidus* spp. |  |
| - All agents | - Susceptibility based on *P. aeruginosa* breakpoint |
| *Elizabethkingia* spp. |  |
| - All agents | - Susceptibility based on *P. aeruginosa* breakpoint |
| *Haemophilus influenzae* |  |
| - All agents | - Susceptible |
| *Moraxella catarrhalis* |  |
| - All agents | - Susceptible |
| *Pseudomonas* spp. |  |
| - Meropenem-vaborbactam | - Susceptibility based on meropenem breakpoint |
| *Stenotrophomonas maltophilia* |  |
| - Cefepime | - Susceptibility based on *P. aeruginosa* breakpoint |
| - Ciprofloxacin | - Susceptibility based on *P. aeruginosa* breakpoint |
| - Ceftazidime-avibactam, ceftolozane-tazobactam, imipenem-relebactam, meropenem-vaborbactam | - Susceptibility based on *P. aeruginosa* breakpoint |

**Supplementary Methods 2.**

| **Antimicrobial stewardship applied to empiric therapy pathways** | |
| --- | --- |
| 1 | While monotherapy was preferred, the widespread use of novel β-lactam agents (i.e., ceftolozane-tazobactam, ceftazidime-avibactam, imipenem-relebactam, meropenem-vaborbactam, and cefiderocol) for all patients in a given unit was not applied as stewardship programs are unlikely to employ such a strategy for a variety of reasons (e.g., resistance concerns, cost, comfort level). Combination therapy was selected if susceptibility rates with monotherapy provided activity in less than 85% of isolates. |
| 2 | If multiple regimens provided similar susceptibility rates, a non-carbapenem β-lactam backbone regimen and/or non-fluoroquinolone-based combinations were preferred. |
| 3 | Tobramycin was the preferred aminoglycoside over amikacin and gentamicin due to the absence of susceptibility breakpoints against *P. aeruginosa* for amikacin and gentamicin for respiratory isolates in the updated 2023 CLSI guidelines.^1^ |
| 4 | The susceptibilities of novel β-lactam agents were also evaluated when traditional combination regimens did not provide activity in at least 85% of isolates.^2^ |

^1^ CLSI. Performance standards for antimicrobial susceptibility testing. 33rd ed. CLSI supplement M100. Clinical and Laboratory Standards Institute; 2023.

^2^ During this study period, susceptibility testing of ceftolozane-tazobactam for Enterobacterales was not performed in our center, which limited its ability to be included in various assessments.

**Supplementary Results 1. A) frequency of Gram-negative bacteria from 221 isolates in period I, B) frequency of Gram-negative bacteria from 159 isolates in period II**

**A)**

| **Gram-negative bacteria** | **All**  **(221 isolates)**  **n (%)** | **Patient-specific risk factor approach** | | | **Unit-specific combination antibiogram approach** | |
| --- | --- | --- | --- | --- | --- | --- |
|  |  | **Without key risk factors**  **(84 isolates)**  **n (%)** | **Recent antibiotic use**  **(100 isolates)**  **n (%)** | **Previous positive resistant-GNB**  **(37 isolates)**  **n (%)** | **MICU**  **(152 isolates)**  **n (%)** | **SICU**  **(69 isolates)**  **n (%)** |
| *Pseudomonas aeruginosa* | 56 (25.3) | 15 (17.9) | 25 (25.0) | 16 (43.2) | 40 (26.3) | 16 (23.2) |
| *Escherichia coli* | 33 (14.9) | 11 (13.1) | 19 (19.0) | 3 (8.1) | 20 (13.2) | 13 (18.8) |
| *Stenotrophomonas maltophilia* | 28 (12.7) | 8 (9.5) | 14 (14.0) | 6 (16.2) | 17 (11.2) | 11 (15.9) |
| *Klebsiella pneumoniae* | 27 (12.2) | 10 (11.9) | 12 (12.0) | 5 (13.5) | 19 (12.5) | 8 (11.6) |
| *Enterobacter cloacae* | 19 (8.6) | 6 (7.1) | 11 (11.0) | 2 (5.4) | 14 (9.2) | 5 (7.2) |
| *Klebsiella oxytoca* | 14 (6.3) | 8 (9.5) | 4 (4.0) | 2 (5.4) | 10 (6.6) | 4 (5.8) |
| *Haemophilus influenzae* | 7 (3.2) | 6 (7.1) | 1 (1.0) | - | 4 (2.6) | 3 (4.3) |
| *Serratia marcescens* | 7 (3.2) | 4 (4.8) | 3 (3.0) | - | 6 (3.9) | 1 (1.4) |
| *Proteus mirabilis* | 6 (2.7) | 6 (7.1) | - | - | 5 (3.3) | 1 (1.4) |
| *Klebsiella aerogenes* | 5 (2.3) | 5 (6.0) | - | - | 4 (2.6) | 1 (1.4) |
| *Citrobacter koseri* | 4 (1.8) | 2 (2.4) | 1 (1.0) | 1 (2.7) | 4 (2.6) | - |
| *Citrobacter freundii* | 2 (0.9) | - | 2 (2.0) | - | 2 (1.3) | - |
| Other Gram-negative | 13 (5.9) | 3 (3.6) | 8 (8.0) | 2 (5.4) | 7 (4.6) | 6 (8.7) |

Abbreviations: GNB, Gram-negative bacteria; MICU, medical intensive care unit; SICU, surgical intensive care unit

**B)**

| **Gram-negative bacteria** | **All**  **(159 isolates)**  **n (%)** | **Patient-specific risk factor approach** | | | **Unit-specific combination antibiogram approach** | |
| --- | --- | --- | --- | --- | --- | --- |
|  |  | **Without key risk factors**  **(60 isolates)**  **n (%)** | **Recent antibiotic use**  **(69 isolates)**  **n (%)** | **Previous positive resistant-GNB**  **(30 isolates)**  **n (%)** | **MICU**  **(117 isolates)**  **n (%)** | **SICU**  **(42 isolates)**  **n (%)** |
| *Pseudomonas aeruginosa* | 51 (32.1) | 17 (28.3) | 20 (29.0) | 14 (46.7) | 41 (35.0) | 10 (23.8) |
| *Stenotrophomonas maltophilia* | 18 (11.3) | 1 (1.7) | 11 (15.9) | 6 (20.0) | 15 (12.8) | 3 (7.1) |
| *Escherichia coli* | 17 (10.7) | 11 (18.3) | 6 (8.7) | - | 11 (9.4) | 6 (14.3) |
| *Klebsiella pneumoniae* | 15 (9.4) | 4 (6.7) | 9 (13.0) | 2 (6.7) | 8 (6.8) | 7 (16.7) |
| *Enterobacter cloacae* | 12 (7.5) | 4 (6.7) | 8 (11.6) | - | 8 (6.8) | 4 (9.5) |
| *Serratia marcescens* | 7 (4.4) | 4 (6.7) | 3 (4.3) | - | 4 (3.4) | 3 (7.1) |
| *Klebsiella aerogenes* | 6 (3.8) | 3 (5.0) | - | 3 (10.0) | 4 (3.4) | 2 (4.8) |
| *Klebsiella oxytoca* | 5 (3.1) | 2 (3.3) | 2 (2.9) | 1 (3.3) | 4 (3.4) | 1 (2.4) |
| *Haemophilus influenzae* | 4 (2.5) | 4 (6.7) | - | - | 4 (3.4) | - |
| *Klebsiella variicola* | 3 (1.9) | 1 (1.7) | 2 (2.9) | - | 3 (2.6) | - |
| *Citrobacter freundii* | 3 (1.9) | - | 2 (2.9) | 1 (3.3) | 2 (1.7) | 1 (2.4) |
| *Achromobacter* species | 3 (1.9) | 2 (3.3) | - | 1 (3.3) | 3 (2.6) | - |
| Other Gram-negative | 15 (4.4) | 7 (11.7) | 6 (8.7) | 2 (6.7) | 10 (8.5) | 5 (11.9) |

Abbreviations: GNB, Gram-negative bacteria; MICU, medical intensive care unit; SICU, surgical intensive care unit

**Supplementary Results 2. Comparisons of different time windows for two risk factors in 190 patients in period I**

| **Risk factor** | **n (%)** |
| --- | --- |
| **Previous positive culture for resistant Gram-negative bacteria** |  |
| - Within 3 months before index culture | 29 (15.3) |
| - Within 1 year before index culture | 32 (16.8) |
| **Previous antibiotic use** |  |
| - Duration ≥ 1 day within 3 months before index culture | 124 (65.3) |
| - Duration ≥ 3 days within 3 months before index culture | 114 (60.0) |
| - Duration ≥ 1 day within 6 months before index culture | 137 (72.1) |
| - Duration ≥ 3 days within 6 months before index culture | 117 (61.6) |
| - Duration ≥ 1 day within 1 year before index culture | 141 (74.2) |
| - Duration ≥ 3 days within 1 year before index culture | 123 (64.7) |

ˠ Defined as having a positive culture from any culture site with a Gram-negative pathogen that displayed resistance to at least one traditional anti-pseudomonal β-lactam (i.e., cefepime, piperacillin-tazobactam, meropenem, or imipenem).

ꚞ Defined as having previous antibiotic use if they had received any of the targeted antibiotics as follows: amikacin, cefepime, cefpodoxime*, ceftazidime, ceftriaxone, ciprofloxacin, ertapenem, gentamicin, imipenem, levofloxacin, meropenem, piperacillin-tazobactam, tobramycin. (Cefpodoxime is the only oral third-generation cephalosporin utilized in study population.)

ꭞ Defined as admitted from an outside healthcare facility if they were transferred from an outside hospital, a skilled nursing facility, or a long-term care facility.

**Supplementary Results 3. Comparison of cumulative susceptibility percentages of Gram-negative respiratory isolates based on admitting location and duration of stay in ICU in patients without previous antibiotic use or previous positive resistant Gram-negative bacteria**


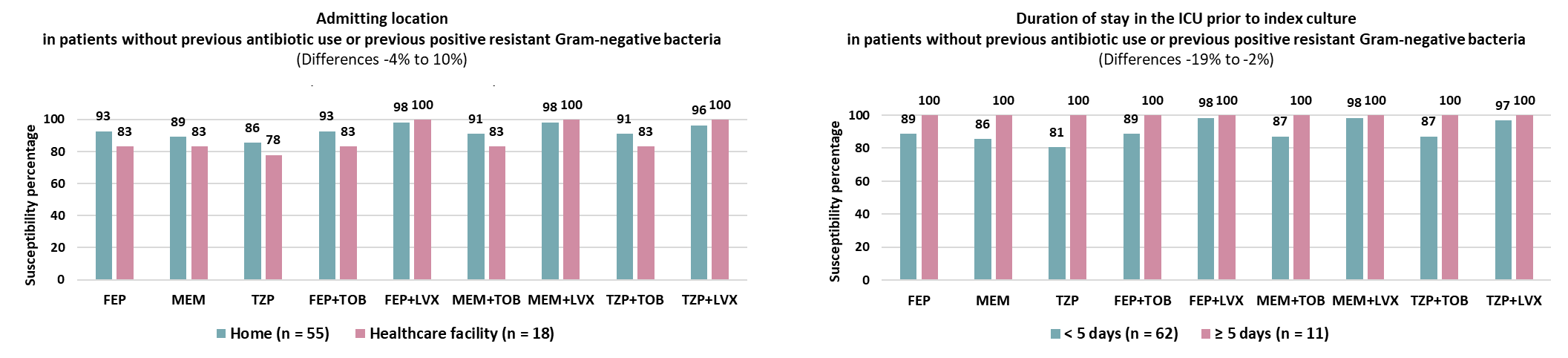


Abbreviations: FEP, Cefepime; MEM, Meropenem; TZP, Piperacillin-tazobactam; TOB, Tobramycin; LVX, Levofloxacin

**Supplementary Results 4.**

| **Selection and rationale for empiric regimens guided by patient-specific risk factors** | | |
| --- | --- | --- |
| **Risk group** | **Empiric regimen selected** | **Rationale** |
| Patients without the two risk factors | Cefepime | - 90% of patients in this subgroup had isolates that demonstrated *in vitro* susceptibility to cefepime. |
| Patients with a history of antibiotic use within 3 months | Cefepime + Levofloxacin | - 86% of patients in this subgroup had isolates that demonstrated *in vitro* susceptibility to this regimen. - While cefepime with tobramycin was considered given its slightly decreased, yet similar (84%) susceptibility rate, this was ultimately not chosen due to both the a priori decision to select a regimen with ≥ 85% activity and the fact that percent susceptibility to tobramycin would decrease with updated 2023 CLSI breakpoints. |
| Patients with a history of resistant Gram-negative bacteria isolation within 1 year | More stratified analysis performed (See Table 1) | - Antimicrobial susceptibilities remained below 85% even when an aminoglycoside or fluoroquinolone was added to the β-lactam backbone. |
| Patients with a previous positive culture for carbapenem-resistant *P. aeruginosa* or Enterobacterales | Cefiderocol | - The highest susceptibility percentage of any combination of traditional agents against patients’ isolates was 75%, and therefore, cefiderocol was selected as it was the only novel agent providing >85% activity. |
| Patients with a previous positive culture with cefepime or piperacillin-tazobactam-resistant P. aeruginosa | Meropenem + Tobramycin | - Meropenem was a significantly more active β-lactam backbone compared to cefepime in this subgroup (80% vs. 40%), combined with the desire to avoid unnecessary fluoroquinolone use. |
| Patients with a previous culture with cefepime or piperacillin-tazobactam resistant Enterobacterales | - Meropenem + Levofloxacin for the subgroups susceptible to fluoroquinolones - Meropenem + Tobramycin for the subgroups resistant to fluoroquinolones | - Further subcategorization based on fluoroquinolone susceptibility of their previous positive isolates allowed more refined recommendations. |

Note: If a patient has a history of different Gram-negative bacteria resistant to the algorithmic recommendation, the recommendation that empiric treatment should be modified to include coverage of that pathogen was made.

**Supplementary Results 5. Comparison of the appropriateness and the overuse of each empiric regimen between two strategies**

| **Empiric regimen** | **Regimen guided by:** | | | | | |
| --- | --- | --- | --- | --- | --- | --- |
|  | **Patient-specific risk factors** | | **Unit-specific combination antibiograms** | | **Sensitivity analysis for**  **unit-specific combination antibiograms** | |
| **Cefepime + Tobramycin** |  | | **Number of patients** | **93** |  | |
|  |  |  | - **Appropriate** | 76 (81.7%) |  | |
|  |  |  | - **Inappropriate** | 17 (18.3%) |  | |
|  |  |  | Resistant *S. maltophilia* (11 pt) | |  | |
|  |  |  | Resistant *Achromobacter* spp. (3 pt) | |  | |
|  |  |  | Resistant *E. coli* (1 pt) | |  | |
|  |  |  | Resistant *P. aeruginosa* (1 pt) | |  | |
|  |  |  | Resistant *P. stuartii* (1 pt) | |  | |
|  |  |  | - **Overuse** | 61 (65.6%) |  | |
| **Cefepime + Levofloxacin** | **Number of patients** | **57** | **Number of patients** | **36** | **Number of patients** | **129** |
|  | - **Appropriate** | 50 (87.7%) | - **Appropriate** | 32 (88.9%) | - **Appropriate** | 112 (86.8%) |
|  | - **Inappropriate** | 7 (12.3%) | - **Inappropriate** | 4 (11.1%) | - **Inappropriate** | 17 (13.2%) |
|  | Resistant *K. pneumoniae* (2 pt) | | Resistant *P. aeruginosa* (2 pt) | | Resistant *P. aeruginosa* (8 pt) | |
|  | Resistant *P. aeruginosa* (2 pt) | | Resistant *K. pneumoniae* (1 pt) | | Resistant *S. maltophilia* (2 pt) | |
|  | Resistant *P. mirabilis* (2 pt) | | Resistant *P. mirabilis* (1 pt) | | Resistant *K. pneumoniae* (2 pt) | |
|  | Resistant *E. coli* (1 pt) | | - **Overuse** | 29 (80.6%) | Resistant *E. coli* (2 pt) | |
|  | - **Overuse** | 36 (63.2%) |  | | Resistant *Proteus mirabilis* (2 pt) | |
|  |  | |  | | Resistant *Achromobacter* spp. (1 pt) | |
|  |  | |  | | - **Overuse** | 90 (69.8%) |
| **Cefepime** | **Number of patients** | **49** |  | |  | |
|  | - **Appropriate** | 45 (91.8%) |  | |  | |
|  | - **Inappropriate** | 4 (8.2%) |  | |  | |
|  | Resistant *E. coli* (2 pt)  Resistant *Achromobacter* spp. (2 pt) | |  | |  | |
|  | - **Overuse** | 0 (0.0) |  | |  | |
| **Cefepime + TMP/SMX** | **Number of patients** | **2** |  | |  | |
|  | - **Appropriate** | 2 (100%) |  | |  | |
|  | - **Overuse** | 0 (0.0) |  | |  | |
| **Meropenem + Tobramycin** | **Number of patients** | **7** |  | |  | |
|  | - **Appropriate** | 5 (71.4%) |  | |  | |
|  | - **Inappropriate** | 2 (28.6%) |  | |  | |
|  | Resistant *S. maltophilia* (1 pt) | |  | |  | |
|  | Resistant *Chryseobacterium* spp. (1 pt) | |  | |  | |
|  | - **Overuse** | 5 (71.4%) |  | |  | |
| **Meropenem + Levofloxacin** | **Number of patients** | **4** |  | |  | |
|  | - **Appropriate** | 4 (100%) |  | |  | |
|  | - **Overuse** | 4 (100%) |  | |  | |
| **Cefiderocol** | **Number of patients** | **10** |  | |  | |
|  | - **Appropriate** | 10 (100%) |  | |  | |
|  | - **Overuse** | 7 (70.0%) |  | |  | |

Abbreviations: pt, patients; TMP/SMX, trimethoprim/sulfamethoxazole
